# The global burden of thyroid cancer and its attributable risk factor in 195 countries and territories: A systematic analysis for the Global Burden of Disease Study

**DOI:** 10.1002/cam4.3970

**Published:** 2021-05-18

**Authors:** Mimi Zhai, Dan Zhang, Jianhai Long, Yi Gong, Fei Ye, Sushun Liu, Yamin Li

**Affiliations:** ^1^ Xiangya Nursing School Central South University Changsha Hunan China; ^2^ Clinical Nursing Teaching and Research Section The Second Xiangya Hospital Central South University Changsha Hunan China; ^3^ Department of Respiratory Beijing Tiantan Hospital Capital Medicine University Beijing China; ^4^ Department of General Surgery The Second Xiangya Hospital Central South University Changsha Hunan China

**Keywords:** ASR, EAPC, GBD study, risk factor, thyroid cancer

## Abstract

**Background:**

Thyroid cancer is a growing threat to human health. Few studies have explored trends of thyroid cancer and relationships with social development factors. In this study, we explored the trend and relationship based on GBD.

**Methods:**

By using GBD study, we obtained detailed data of thyroid cancer. Incidence, mortality and DALY were used to assess epidemiological characteristics. ASR and EAPC were used to estimate the trend.

**Results:**

Globally, the incidence significantly increased from 1990 to 2017, especially in high‐income regions. Males and middle SDI region demonstrated a higher increase of age‐standardized incidence rates. Unlike incidence trend, mortality trend showed a minor increase, and even showed a decreasing trend in some regions such as Eastern Sub‐Saharan Africa. Additionally, the DALY trend also demonstrated a slightly increase with an EAPC of 0.77 (95% CI 0.73–0.81). More significant increase of DALY was found in males, middle SDI region and high‐income Asia Pacific. The incidence of thyroid cancer peaked in middle‐aged people, while the mortality and DALY peaked in elder‐aged. Moreover, the proportion of thyroid cancer deaths contributable to high BMI was highest in developed countries and middle‐aged people.

**Conclusions:**

Thyroid cancer is a public health problem worldwide. Over‐diagnosis might be partly responsible for its rising trend. It remains us to revise the guidelines to avoid unnecessary burdens. Moreover, we should pay attention to the risk factors of thyroid cancer. More targeted measures should be formulated to improve potential environmental and lifestyle‐related factors which might contribute to rising trend of thyroid cancer.

## Graphical abstract

Our study shows the incidence, mortality and DALY of thyroid cancer are on the rise, and the incidence reaches its peak in middle‐aged people, while the mortality and DALY reach its peak in elderly. The proportion of deaths contributable to high BMI is highest in developed countries and middle‐aged people. In conclusion, these results suggest us to revise guidelines to avoid unnecessary burdens, and formulate more targeted measures to improve environment and lifestyle‐related factors.

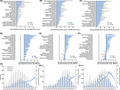

## 1 INTRODUCTION

Thyroid cancer is an endocrine system tumor with high incidence.^1^ Although causes of thyroid cancer are still unclear, ionizing radiation has been proved to be a risk factor.^2^ With the increasing incidence, the incidence of thyroid cancer has ranked 9th among all cancers. In 2016, there were 238,000 thyroid cancer cases worldwide, while the number of patients with thyroid cancer had increased 1.28 times with 567,000 cases only in 2 years.^3^, ^4^ In addition, the burdens of thyroid cancer accounted for 5.1% among all cancers in women.^4^

The Global Burden of Disease (GBD) is a comprehensive and systematic study of health losses for capturing the burden and trend of disease and injury.^5^, ^6^ By analyzing the GBD study, we can explore the changing health challenges. At present, Few studies were published by analyzing GBD data of thyroid cancer.^2^ Additionally, there is no correlation study between sociological indicators (such as socio‐demographic index (SDI), human development index (HDI)) and thyroid cancer epidemic trend.^7^ The aim of this study is to explore the current trend around the world during the 27 year of the incidence, death, and disability adjusted life year (DALY) of thyroid cancer. Moreover, the risk factor of thyroid cancer obtained from GBD study is also study. Our study will provide a basis for healthy policy makers to comprehend the burden of thyroid cancer worldwide, and formulate targeted prevention and intervention measures.

## 2 METHODS

### 2.1 Study data

The data of thyroid cancer by sex, age, regions and countries was obtained from GBD 2017 study. The risk factors of thyroid cancer were collected from the GBD database. Data was collected and analyzed via Global Health Data Exchange query tool. Geographically, the 195 countries and territories were divided into 21 regions according geographical position. In addition, all the countries were grouped as low, low‐middle, middle, high‐middle, and high SDI regions according to the SDI data.

### 2.2 Statistical analysis

Incidence, mortality and DALY were used to assess the epidemiological features of thyroid cancer. In addition, age‐standardized rates (ASR) and estimating the annual percentage change (EAPC) were also introduced to assess trends of thyroid cancer. The calculation of the ASR and EAPC were similar with Liu *et al* study.^8^ By analyzing ASR, we could understand the incidence, death and DALY of thyroid cancer and its changes. EAPC was used to measure the trend of ASR during a period. By using ASR and EAPC, more targeted strategies could be established. In addition, relationship between SDI, HDI and ASR, EAPC was studied to explore the influential factors for ASR and EAPC of thyroid cancer. Furthermore, we visualized the ASR and EAPC of thyroid cancer deaths attributable to risk factor at national levels for 195 countries and territories. R software (Institute for Statistics and Mathematics) and STATA (StataCorp LLC) were used to analyze the data and estimate the trend. A *p*‐value less than 0.05 was considered statistically significant.

## 3 RESULTS

### 3.1 General epidemic trend of thyroid cancer

The incident cases of thyroid cancer were 255,490 in 2017, which was 2.69 times than that of 1990. The age‐standardized incidence rates were 3.34 per 100,000 people in 2017 and demonstrated an increase trend with an EAPC of 2.52 (95% CI 2.44–2.60) during 27 years (Table 1, Figure S1). A total of 41,240 cases died of thyroid cancer in 2017, an increase of 86.86% compared to that of 1990. The age‐standardized death rates were 0.54 per 100,000 people in 2017, which increased from 0.41 in 1990. Similar with the trend of age‐standardized incidence rates, the age‐standardized death rates showed an upward trend with the EAPC of 1.06 (95% CI 0.99–1.12) (Table 2, Figure S2). Additionally, thyroid cancer caused 1.13 million DALYs in 2017. The age‐standardized DALY rates demonstrated a minor increase trend, and the EAPC of DALY was 0.77 (95% CI 0.73–0.81) (Table 3, Fig S3).

**TABLE 1:** The incident cases, age‐standardized incidence rates, and temporal trend of thyroid cancer

Characteristics	1990		2017		1990–2017
	Incident cases No. ×10^3^ (95% UI)	ASR per 100,000 No. (95% UI)	Incident cases No. ×10^3^ (95% UI)	ASR per 100,000 No. (95% UI)	EAPC No. (95% CI)
Overall	95.03 (90.07–100.72)	1.76 (1.67–1.87)	255.49 (245.71–272.47)	3.34 (3.22–3.57)	2.52 (2.44–2.60)
Sex					
Male	24.17 (23.33–25.21)	0.89 (0.86–0.93)	76.09 (72.58–79.29)	1.98 (1.89–2.07)	3.26 (3.15–3.36)
Female	70.85 (66.09–76.42)	2.65 (2.47–2.85)	179.40 (170.40–195.54)	4.71 (4.48–5.14)	2.23 (2.16–2.31)
Socio‐demographic index					
Low	5.71 (3.97–7.39)	0.82 (0.57–1.06)	15.40 (13.73–17.48)	1.19 (1.06–1.36)	1.34 (1.21–1.48)
Low‐middle	9.65 (8.29–11.62)	0.92 (0.79–1.11)	31.54 (28.46–36.75)	1.85 (1.67–2.16)	2.60 (2.52–2.67)
Middle	15.47 (14.50–17.77)	1.00 (0.93–1.15)	60.93 (57.10–69.46)	2.92 (2.73–3.32)	4.13 (4.04–4.23)
Middle‐high	20.99 (19.08–22.13)	1.89 (1.72–1.99)	58.68 (55.41–62.29)	4.23 (3.99–4.49)	3.05 (2.88–3.21)
High	42.95 (42.08–43.83)	4.45 (4.36–4.54)	88.07 (85.08–91.93)	7.73 (7.46–8.07)	2.34 (2.09–2.59)
Region					
Oceania	0.07 (0.05–0.08)	1.01 (0.83–1.24)	0.19 (0.14–0.23)	1.47 (1.15–1.86)	1.38 (1.23–1.54)
Andean Latin America	0.42 (0.37–0.49)	1.09 (0.97–1.27)	2.35 (2.02–2.68)	3.82 (3.29–4.36)	5.11 (4.69–5.53)
Australasia	0.64 (0.60–0.69)	3.18 (2.95–3.42)	2.03 (1.77–2.31)	7.13 (6.23–8.15)	3.58 (3.38–3.77)
Caribbean	0.56 (0.52–0.60)	1.58 (1.47–1.71)	1.44 (1.30–1.60)	3.12 (2.81–3.45)	2.65 (2.43–2.86)
Central Asia	1.02 (0.93–1.16)	1.46 (1.33–1.66)	1.74 (1.61–1.90)	1.92 (1.77–2.09)	0.66 (0.21–1.11)
Central Europe	4.81 (4.62–5.01)	3.88 (3.72–4.04)	6.50 (6.11–6.95)	5.66 (5.32–6.05)	1.34 (1.19–1.48)
Central Latin America	2.03 (1.96–2.11)	1.24 (1.19–1.28)	8.52 (8.09–9.03)	3.33 (3.17–3.54)	3.61 (3.50–3.72)
Central Sub‐Saharan Africa	0.20 (0.15–0.27)	0.37 (0.28–0.49)	0.48 (0.38–0.68)	0.39 (0.32–0.56)	0.02 (−0.19–0.24)
East Asia	12.09 (10.54–13.22)	0.96 (0.84–1.05)	44.77 (41.55–50.71)	3.01 (2.80–3.41)	4.35 (4.02–4.68)
Eastern Europe	7.85 (7.25–8.78)	3.46 (3.20–3.87)	14.50 (13.61–15.53)	6.90 (6.47–7.39)	2.69 (2.39–2.99)
Eastern Sub‐Saharan Africa	1.99 (1.34–2.64)	1.04 (0.70–1.38)	4.59 (3.91–5.46)	1.17 (0.99–1.39)	0.18 (−0.02–0.39)
High‐income Asia Pacific	6.68 (6.40–7.02)	3.85 (3.69–4.04)	20.62 (18.89–22.71)	11.02 (10.10–12.14)	5.17 (4.51–5.83)
High‐income North America	11.98 (11.67–12.32)	4.27 (4.16–4.39)	28.28 (27.19–29.35)	7.84 (7.53–8.13)	2.17 (1.95–2.39)
North Africa and Middle East	3.56 (2.89–4.41)	1.04 (0.85–1.29)	17.47 (15.99–19.89)	2.91 (2.66–3.31)	4.17 (4.06–4.29)
South Asia	10.54 (8.75–12.91)	0.95 (0.79–1.16)	37.97 (33.68–43.03)	2.13 (1.89–2.41)	3.07 (2.92–3.22)
Southeast Asia	6.84 (5.86–7.84)	1.46 (1.26–1.68)	23.88 (21.43–29.12)	3.62 (3.24–4.41)	3.48 (3.45–3.51)
Southern Latin America	1.10 (1.03–1.18)	2.22 (2.08–2.38)	2.31 (2.09–2.59)	3.53 (3.18–3.95)	1.54 (1.34–1.74)
Southern Sub‐Saharan Africa	0.39 (0.33–0.43)	0.74 (0.62–0.82)	0.76 (0.68–0.86)	0.98 (0.88–1.11)	0.62 (0.12–1.12)
Tropical Latin America	1.83 (1.75–1.91)	1.19 (1.14–1.24)	5.54 (5.27–5.77)	2.53 (2.41–2.64)	2.81 (2.61–3.01)
Western Europe	19.90 (19.19–20.63)	5.16 (4.98–5.35)	30.26 (28.58–32.03)	6.99 (6.60–7.40)	1.20 (1.01–1.39)
Western Sub‐Saharan Africa	0.53 (0.41–0.61)	0.28 (0.21–0.32)	1.30 (1.11–1.56)	0.30 (0.26–0.36)	0.19 (0.07–0.31)

**TABLE 2:** The death cases, age‐standardized death rates, and temporal trend of thyroid cancer

Characteristics	1990		2017		1990–2017
	Death cases No. ×10^3^ (95% UI)	ASR per 100,000 No. (95% UI)	Death cases No. ×10^3^ (95% UI)	ASR per 100,000 No. (95% UI)	EAPC No. (95% CI)
Overall	22.07 (20.81–24.22)	0.41 (0.39–0.45)	41.24 (39.91–44.14)	0.54 (0.52–0.58)	1.06 (0.99–1.12)
Sex					
Male	7.56 (7.22–8.15)	0.28 (0.27–0.30)	17.16 (16.41–17.77)	0.45 (0.43–0.46)	1.97 (1.86–2.07)
Female	14.51 (13.46–16.77)	0.54 (0.50–0.63)	24.08 (23.06–26.83)	0.63 (0.61–0.71)	0.49 (0.44–0.55)
Socio‐demographic index					
Low	2.40 (1.81–3.00)	0.34 (0.26–0.43)	4.53 (4.06–5.01)	0.35 (0.31–0.39)	0.03 (−0.08–0.13)
Low‐middle	3.33 (2.96–3.95)	0.32 (0.28–0.38)	7.27 (6.77–8.22)	0.43 (0.40–0.48)	1.04 (1.00–1.09)
Middle	4.85 (4.57–5.73)	0.31 (0.29–0.37)	11.61 (11.04–12.89)	0.56 (0.53–0.62)	2.28 (2.15–2.40)
Middle‐high	4.51 (4.29–4.72)	0.41 (0.39–0.42)	7.66 (7.34–8.05)	0.55 (0.53–0.58)	1.12 (1.01–1.22)
High	6.90 (6.81–6.99)	0.71 (0.71–0.72)	10.02 (9.71–10.38)	0.88 (0.85–0.91)	0.81 (0.77–0.86)
Region					
Oceania	0.02 (0.02–0.03)	0.36 (0.30–0.44)	0.05 (0.04–0.06)	0.40 (0.33–0.49)	0.42 (0.38–0.46)
Andean Latin America	0.16 (0.15–0.18)	0.42 (0.38–0.47)	0.53 (0.46–0.58)	0.86 (0.75–0.95)	2.96 (2.73–3.20)
Australasia	0.09 (0.09–0.09)	0.44 (0.42–0.46)	0.19 (0.17–0.21)	0.68 (0.61–0.75)	2.09 (1.89–2.28)
Caribbean	0.14 (0.13–0.15)	0.39 (0.36–0.43)	0.27 (0.25–0.29)	0.58 (0.53–0.63)	1.56 (1.33–1.79)
Central Asia	0.21 (0.20–0.24)	0.31 (0.29–0.35)	0.26 (0.25–0.28)	0.29 (0.27–0.30)	−0.75 (−1.03‐−0.46)
Central Europe	1.07 (1.04–1.09)	0.86 (0.84–0.88)	0.92 (0.88–0.96)	0.80 (0.77–0.84)	−0.52 (−0.73‐−0.32)
Central Latin America	0.61 (0.59–0.63)	0.37 (0.36–0.38)	1.56 (1.50–1.64)	0.61 (0.59–0.64)	1.81 (1.61–2.01)
Central Sub‐Saharan Africa	0.10 (0.08–0.13)	0.18 (0.15–0.23)	0.19 (0.15–0.25)	0.15 (0.12–0.20)	−0.81 (−0.93‐−0.68)
East Asia	3.32 (3.10–3.87)	0.26 (0.25–0.31)	7.25 (6.83–7.92)	0.49 (0.46–0.53)	2.58 (2.26–2.91)
Eastern Europe	1.34 (1.29–1.45)	0.59 (0.57–0.64)	1.66 (1.61–1.73)	0.79 (0.77–0.82)	0.80 (0.41–1.19)
Eastern Sub‐Saharan Africa	0.88 (0.63–1.16)	0.46 (0.33–0.61)	1.41 (1.21–1.64)	0.36 (0.31–0.42)	−1.21 (−1.38‐−1.04)
High‐income Asia Pacific	1.14 (1.12–1.16)	0.66 (0.64–0.67)	2.77 (2.65–2.94)	1.48 (1.42–1.57)	3.39 (3.11–3.67)
High‐income North America	1.30 (1.27–1.32)	0.46 (0.45–0.47)	2.38 (2.30–2.45)	0.66 (0.64–0.68)	1.31 (1.27–1.34)
North Africa and Middle East	0.86 (0.72–1.07)	0.25 (0.21–0.31)	1.92 (1.80–2.28)	0.32 (0.30–0.38)	0.96 (0.88–1.04)
South Asia	3.63 (3.14–4.39)	0.33 (0.28–0.40)	8.93 (8.18–9.84)	0.50 (0.46–0.55)	1.61 (1.52–1.70)
Southeast Asia	2.33 (2.01–2.74)	0.50 (0.43–0.59)	4.82 (4.46–5.70)	0.73 (0.67–0.86)	1.45 (1.42–1.47)
Southern Latin America	0.32 (0.31–0.33)	0.64 (0.62–0.67)	0.43 (0.40–0.47)	0.65 (0.60–0.71)	−0.06 (−0.25–0.14)
Southern Sub‐Saharan Africa	0.11 (0.10–0.13)	0.22 (0.19–0.24)	0.22 (0.20–0.24)	0.28 (0.26–0.31)	0.96 (0.46–1.45)
Tropical Latin America	0.53 (0.52–0.55)	0.35 (0.34–0.36)	1.13 (1.09–1.16)	0.52 (0.50–0.53)	1.48 (1.43–1.53)
Western Europe	3.62 (3.56–3.69)	0.94 (0.92–0.96)	3.86 (3.68–4.05)	0.89 (0.85–0.93)	−0.25 (−0.32‐−0.19)
Western Sub‐Saharan Africa	0.27 (0.21–0.32)	0.14 (0.11–0.17)	0.48 (0.41–0.55)	0.11 (0.09–0.13)	−1.18 (−1.30‐−1.06)

**TABLE 3:** The DALY cases, age‐standardized DALY rates, and temporal trend of thyroid cancer

	1990		2017		1990–2017
Characteristics	DALYs No. ×10^3^ (95% UI)	ASR per 100,000 No. (95% UI)	DALYs No. ×10^3^ (95% UI)	ASR per 100,000 No. (95% UI)	EAPC No. (95% CI)
Overall	648.24 (595.58–713.24)	12.02 (11.04–13.22)	1133.17 (1073.44–1227.49)	14.83 (14.05–16.07)	0.77 (0.73–0.81)
Sex					
Male	226.93 (213.37–247.06)	8.35 (7.85–9.09)	468.25 (444.42–492.88)	12.21 (11.59–12.85)	1.55 (1.47–1.63)
Female	421.31 (375.69–489.01)	15.74 (14.03–18.27)	664.93 (618.50–746.73)	17.47 (16.25–19.62)	0.28 (0.24–0.32)
Socio‐demographic index					
Low	89.84 (63.65–114.09)	12.88 (9.12–16.36)	153.32 (136.63–169.90)	11.89 (10.59–13.17)	−0.41 (−0.47‐−0.35)
Low‐middle	115.39 (100.21–134.86)	11.05 (9.60–12.92)	236.77 (214.78–270.13)	13.89 (12.60–15.85)	0.79 (0.72–0.86)
Middle	148.92 (139.53–172.28)	9.60 (9.00–11.11)	314.77 (294.66–346.94)	15.06 (14.10–16.60)	1.74 (1.62–1.86)
Middle‐high	129.18 (119.94–136.17)	11.62 (10.79–12.25)	204.21 (190.73–220.65)	14.72 (13.75–15.90)	0.75 (0.63–0.88)
High	162.78 (155.31–171.62)	16.85 (16.08–17.77)	220.44 (205.12–239.51)	19.34 (18.00–21.01)	0.63 (0.53–0.72)
Region					
Oceania	0.77 (0.64–0.96)	11.91 (9.96–14.79)	1.67 (1.36–2.13)	13.22 (10.78–16.87)	0.46 (0.42–0.51)
Andean Latin America	4.49 (4.03–5.07)	11.71 (10.51–13.22)	12.82 (11.19–14.27)	20.86 (18.21–23.22)	2.41 (2.16–2.66)
Australasia	2.28 (2.14–2.45)	11.27 (10.53–12.10)	4.77 (4.17–5.44)	16.80 (14.70–19.17)	1.96 (1.78–2.14)
Caribbean	3.80 (3.41–4.23)	10.77 (9.66–11.97)	7.07 (6.36–7.87)	15.28 (13.75–17.02)	1.40 (1.17–1.63)
Central Asia	6.63 (6.17–7.50)	9.50 (8.85–10.75)	7.63 (7.09–8.23)	8.39 (7.79–9.05)	−1.01 (−1.29‐−0.73)
Central Europe	27.87 (26.83–29.07)	22.45 (21.62–23.42)	22.23 (20.81–23.89)	19.36 (18.13–20.81)	−0.85 (−1.05‐−0.65)
Central Latin America	16.48 (15.91–17.09)	10.04 (9.69–10.41)	39.54 (37.35–42.02)	15.48 (14.62–16.45)	1.55 (1.35–1.75)
Central Sub‐Saharan Africa	3.14 (2.43–4.07)	5.70 (4.41–7.39)	5.74 (4.64–7.68)	4.72 (3.82–6.31)	−0.89 (−1.02‐−0.76)
East Asia	104.01 (96.07–117.48)	8.26 (7.63–9.33)	184.14 (170.57–203.49)	12.39 (11.48–13.70)	1.52 (1.16–1.88)
Eastern Europe	35.79 (33.65–39.61)	15.77 (14.83–17.46)	44.72 (41.69–48.39)	21.28 (19.83–23.02)	0.82 (0.42–1.22)
Eastern Sub‐Saharan Africa	34.05 (22.78–45.82)	17.77 (11.89–23.92)	52.65 (44.75–61.71)	13.39 (11.38–15.69)	−1.36 (−1.53‐−1.20)
High‐income Asia Pacific	25.92 (24.64–27.50)	14.94 (14.20–15.85)	52.27 (47.68–57.63)	27.95 (25.49–30.81)	3.04 (2.62–3.46)
High‐income North America	33.04 (30.97–35.48)	11.77 (11.03–12.64)	60.84 (56.16–66.36)	16.86 (15.56–18.39)	1.30 (1.23–1.37)
North Africa and Middle East	27.73 (22.70–33.94)	8.14 (6.66–9.96)	63.08 (57.86–72.30)	10.51 (9.64–12.05)	1.05 (0.99–1.11)
South Asia	135.98 (114.47–164.22)	12.26 (10.32–14.81)	297.71 (269.76–332.09)	16.70 (15.13–18.63)	1.15 (1.06–1.23)
Southeast Asia	71.02 (60.63–81.06)	15.22 (12.99–17.37)	134.52 (122.89–156.20)	20.37 (18.61–23.65)	1.10 (1.08–1.13)
Southern Latin America	8.00 (7.63–8.36)	16.15 (15.40–16.88)	10.04 (9.12–11.10)	15.30 (13.90–16.92)	−0.33 (−0.56‐−0.10)
Southern Sub‐Saharan Africa	3.50 (3.08–3.84)	6.68 (5.87–7.31)	6.32 (5.74–7.01)	8.17 (7.42–9.06)	0.73 (0.12–1.35)
Tropical Latin America	14.83 (14.29–15.48)	9.67 (9.31–10.09)	28.57 (27.18–30.04)	13.06 (12.43–13.73)	1.11 (1.04–1.19)
Western Europe	80.85 (77.15–84.69)	20.96 (20.00–21.96)	82.73 (76.83–90.09)	19.11 (17.74–20.81)	−0.36 (−0.42‐−0.31)
Western Sub‐Saharan Africa	8.05 (6.31–9.21)	4.19 (3.28–4.79)	14.10 (12.27–16.32)	3.25 (2.83–3.76)	−1.17 (−1.30‐−1.04)

### 3.2 Epidemiological characteristics and estimate trends of thyroid cancer by regions, sex, and age

**FIGURE 1:**
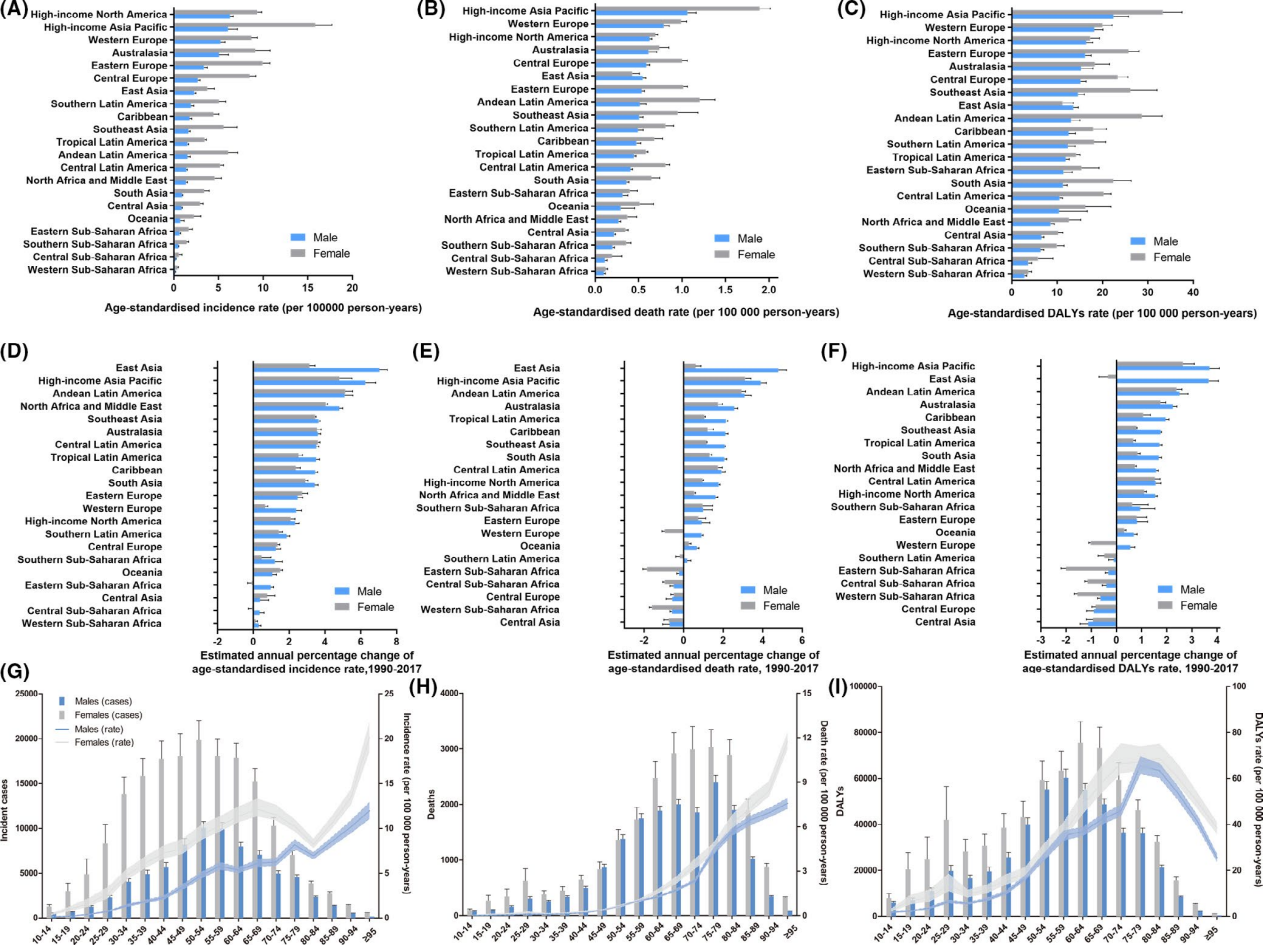
The trend of thyroid cancer incidence, death, and DALY for 21 GBD regions by sex and age. The age‐standardized incidence (A), death(B), and DALY(C) rates of thyroid cancer by sex. The EAPC of age‐standardized incidence (D), death(E), and DALY(F) rates of thyroid cancer for 21 regions by sex from 1990 to 2017. Age‐specific counts and rate of incident cases (G), deaths (H), and DALYs (I) by sex in 2017

In 2017, age‐standardized incidence rates were higher in females (Figure 1A). Similarly, age‐standardized death and DALY rates were also higher in females except for East Asia (Figure 1B,C). Among all regions, high‐income North America, high‐income Asia Pacific and Western Europe were found to obtain the highest age‐standardized rates of incidence, death and DALY in 2017 (Figure 1A–C). For geographical regions, the age‐standardized rates of incidence, death and DALY of thyroid cancer in the South Korea and Iceland were the highest (Figure S1–S3). Contrary, the lowest age‐standardized rates of incidence, death and DALY were observed in Sub‐Saharan Africa regions in 2017 (Figure S1–S3, Tables 1, 2, 3).

The high‐income Asia Pacific, East Asia, and Andean Latin America had the highest EAPC of incidence, death, and DALY (Figure 1D–F, Tables 1, 2, 3). It was worth noting that the trend of age‐standardized incidence rates of women in Eastern Sub‐Saharan Africa and Central Sub‐Africa presented a decrease trend (Figure 1D). In addition, Eastern Sub‐Saharan Africa, Central Sub‐Saharan Africa, Central Europe, Western Sub‐Saharan Africa and Central Asia demonstrated a decrease trend of age‐standardized death and DALY rates both in males and females (Figure 1E,F). Although high‐income North America and Western Europe had the highest age‐standardized rates of incidence, death and DALY in 2017, the trend of them in these countries only demonstrated a minor increase from 1990 to 2017 (Figure 1D,E). Amazingly, the age‐standardized incidence, death and DALY rates had dropped most obviously in Sub‐Saharan Africa regions, especially in Western Sub‐Saharan Africa (Tables 1, 2, 3, Figure S1–S3).

In terms of age, the incident cases peaked in middle‐aged people, especially between 50 and 54 years old group. The age‐standardized incidence rates gradually increased with age, except for the cases in the age of 80–84 years old (Figure 1G). The number of deaths was higher in elderly people aged from 65 to 84 years old, and the age‐standardized death rates gradually increased with age in all age grades (Figure 1H). In addition, the number of deaths in female was higher than males, except for the females aged from 45 to 59 years old (Figure 1H). Though the number of DALY cases was also higher in the elderly, the age‐standardized DALY rates declined after reaching its highest value in the age range of 70–79 years old in females and males (Figure 1I).

### 3.3 Trend of thyroid cancer deaths attributable to risk factor by regions, sex, and age

**FIGURE 2:**
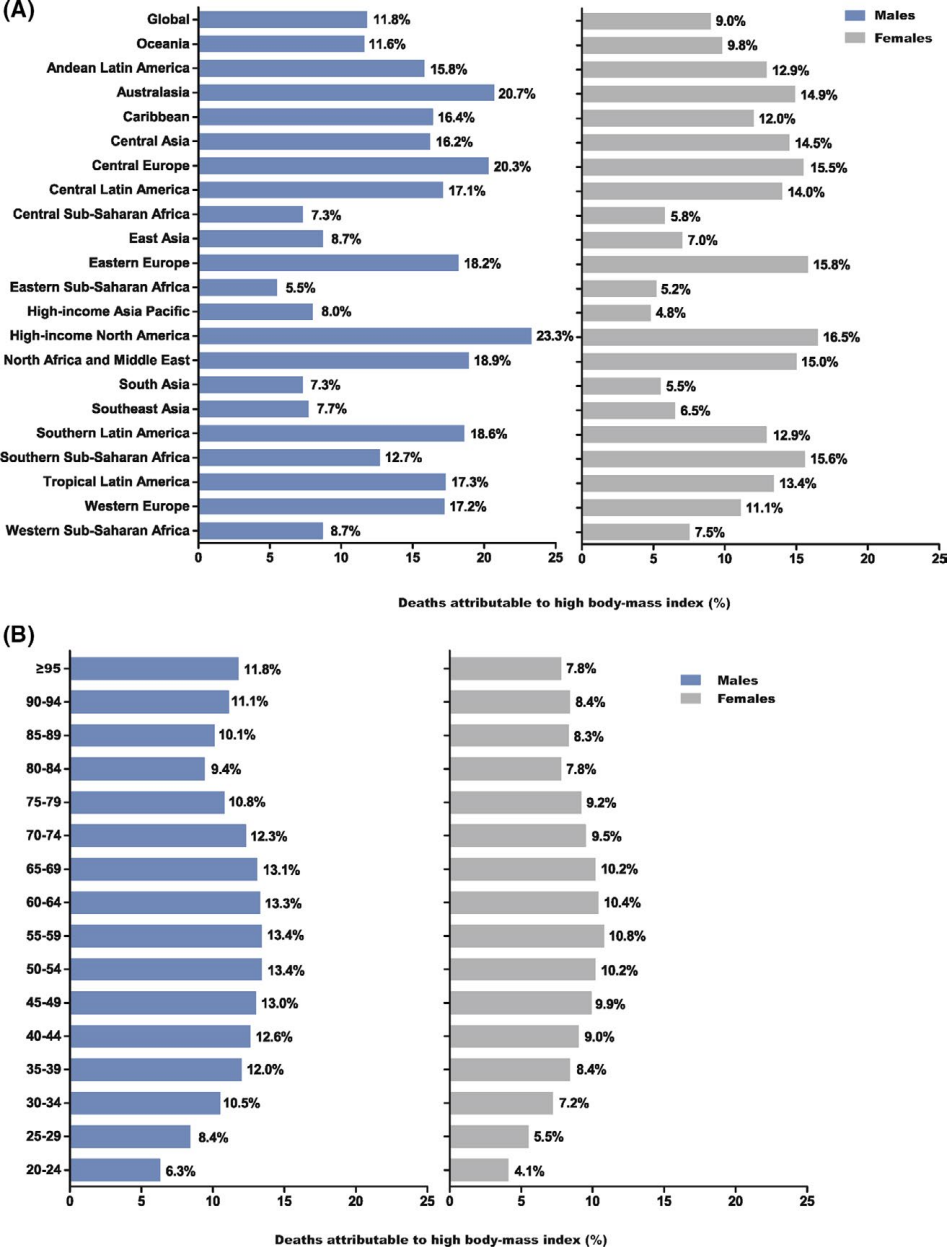
Fraction of thyroid cancer age‐standardized deaths attributable to high BMI by regions (A) and age groups (B) for males and females in 2017

The percentage of deaths of thyroid cancer attributable to high body‐mass index (BMI) was shown in Figure 2 and it varied from country to country. Globally, 11.8% of thyroid cancer deaths in female was attributable to high BMI, and was higher compared with that in males (Figure 2A). However, it should be noted that deaths attributable to high BMI in males was highest in high‐income North America (23.3%), followed by Australasia (20.7%). Additionally, it was also the highest in high‐income North America (16.5%) in females, followed by Eastern Europe (15.8%)(Figure 2A). After grouping by age range, we found that it was highest in people aged 55–59 both in males and females, and was lowest in people aged 20–24 years old (Figure 2B).

### 3.4 The influential factors for ASR and EAPC of incidence, death, and DALY

**FIGURE 3:**
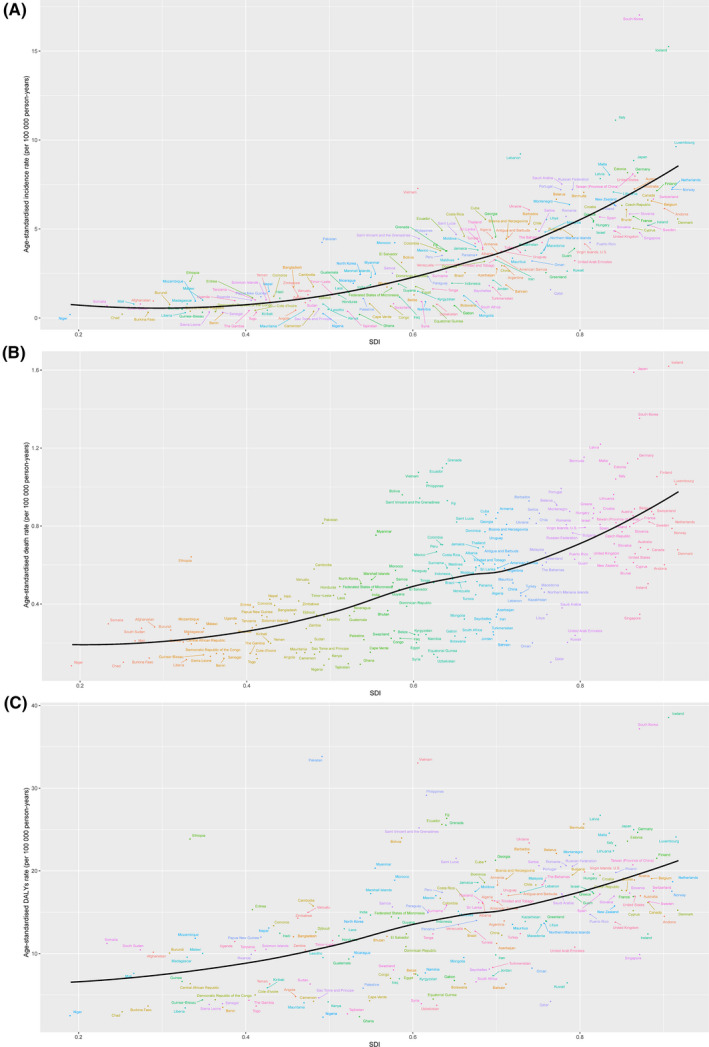
The age‐standardized incidence (A), death (B), and DALY rates of thyroid cancer across 195 countries and territories by SDI in 2017

An increase trend of age‐standardized incidence rates along with increase of SDI was found in our study (Figure 3A). The observed levels were much higher in South Korea and Iceland compared with expected levels (Figure 3A). Additionally, a similar correlation was found between age‐standardized death rates, age‐standardized DALY rates and SDI (Figure 3B,C). Similarly, the observed levels were much higher in Iceland, Japan and South Korea (Figure 3B,C). Additionally, we visualized the trend in age‐standardized incidence rates across SDI (Figure S4). Incidence rates in regions generally had maintained a trend of simultaneous growth with SDI(Figure S4A). Among all the regions, only high‐income Asia Pacific demonstrated a sharp decrease in age‐standardized incidence rates, but not down to 1990 levels (Figure S4A). Although Central, Southern, and Western Sub‐Saharan Africa had rising age‐standardized incidence rates, the value of it in these countries was the lowest. In addition, the age‐standardized incidence rates of these countries were below the expected levels in all years (Figure S4A). Moreover, high‐income regions had a significant growth in age‐standardized incidence rates (Figure S4A). We also visualized the trend in age‐standardized rates of death and DALY across SDI from 1990 to 2017 (Figure S4B,C). Similar results could be obtained (Figure S4B,C).

**FIGURE 4:**
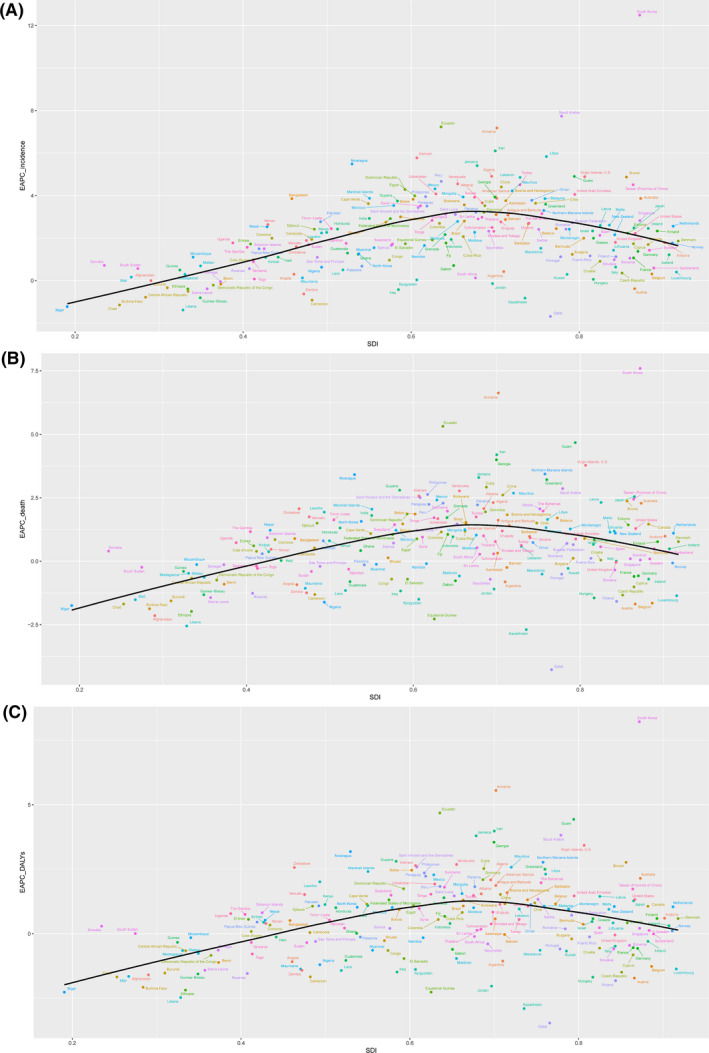
The EAPC of age‐standardized incidence (A), death (B), and DALY rates of thyroid cancer across 195 countries and territories by SDI from 1990 to 2017

Correlation analysis between SDI and EAPC was also conducted in our study. We found that SDI was positively correlated with EAPC of incidence, death and DALY when SDI was less than 0.691, 0.682, and 0.681, respectively. Additionally, SDI was negatively correlated with EAPC when SDI greater than the cut‐off (Figure 4A–C). We also studied the relationship between age‐standardized rates and HDI in 2017 (Figure S5). An increase trend of age‐standardized rates of incidence, death, and DALY along with increase of SDI was found in our study (Figure S5).

### 3.5 The ASR and EAPC of death attributable to risk factor by countries and territories

**FIGURE 5:**
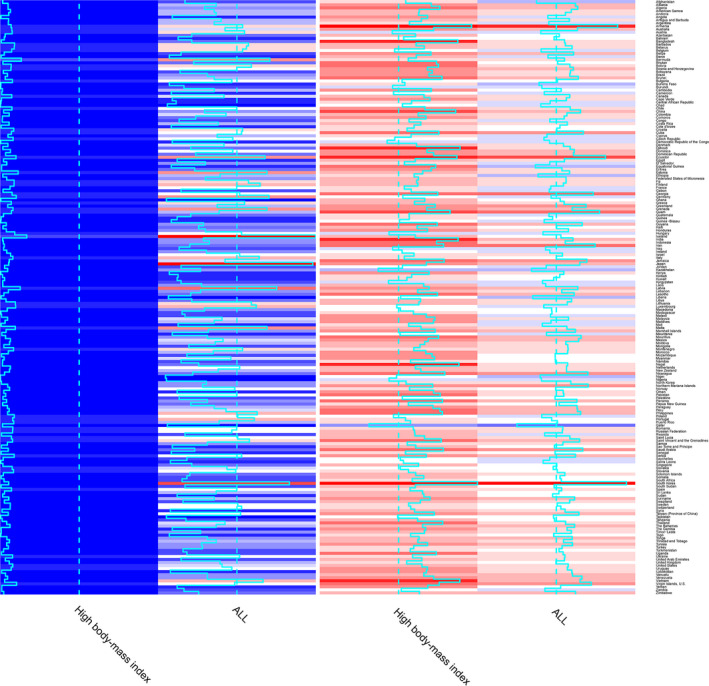
Heatmaps for ASR and EAPCs on risk factor of thyroid cancer in 195 countries and territories in both sexes in 2017

The EAPC of thyroid cancer deaths caused by high BMI was less than 0 in 16 countries, and indicated a downward trend. Among the countries, Qatar, Kazakhstan, and Democratic Republic of the Congo were found to have the lowest EAPC of death caused by high BMI(Figure 5). In contrast, the countries with the highest EAPC of death caused by high BMI were South Korea, Armenia, and Djibouti. The countries with the highest ASR of thyroid cancer deaths caused by high BMI were Iceland, Japan, and South Korea (Figure 5).

## 4 DISCUSSION

Thyroid cancer is a common malignant tumor of the endocrine system.^4^ The morbidity and mortality are still increasing worldwide.^9^, ^10^ Previous studies explored the incidence and mortality by using outdated data of thyroid cancer.^11^, ^12^, ^13^ In our study, we demonstrated key findings and standpoints of thyroid cancer based on GBD​ 2017 study. In the study, we comprehensively estimated the burden and trends of thyroid cancer, explored the correlation between thyroid cancer and sociological indicators, and studied the deaths attributable to risk factor. In general, all of the thyroid cancer incidence, mortality and DALY demonstrated an increase trend during the study period. High BMI, a risk factor obtained from GBD study, was associated with 11.8% of female deaths and 9.0% of male deaths caused by high BMI among all deaths. Thus, we should pay more attention to recognize the risk factors of thyroid cancer.

In our study, we found that many countries obtained an increasing incidence of thyroid cancer, including high‐income regions. In addition, ASR demonstrated a growth trend in all regions except for Central and Eastern Sub‐Saharan Africa. The rising incidence seemed to be related with the growing use of imaging examination, ultrasonic examination and biopsy. By using these new examination technologies, detection and diagnosis of early stage thyroid cancer had increased significantly.^14^, ^15^ Thus, update of thyroid cancer detection technologies undoubtedly accounted for at least some of the increasing incidence.^14^, ^16^, ^17^ By fully understanding the contribution of over‐diagnosis to thyroid cancer, relevant institutions, such as American Thyroid Association (ATA), had made stricter restrictions on the indications for thyroid nodule biopsy.^18^, ^19^ By gradually recognizing the over‐diagnosis on a global scale, the increase trend of incidence might be reversed in few decades. Moreover, the EAPC of incidence in males were higher compared to females, especially in East Asia and high‐income Asia Pacific. It might be related with the promotion of screening program for cancer in these countries. Moreover, men suffered with thyroid cancer, which was not detected before had been diagnosed in recent years.^10^, ^20^ Thus, we suspected that EAPC of thyroid cancer incidence in men would gradually decrease, and might be lower than that in women in few decades.

Unlike the rising incidence, the mortality of thyroid cancer in most countries and territories demonstrated a minor increased or stable trend during the period. Among all regions, a higher EAPC of deaths was found in high‐income region. It was worth noting that the mortality of thyroid cancer did not increase with the significant increase in incidence. It indicated that over‐diagnosis was not the only explanation.^21^ Other risk factors also might be related with the incidence and mortality of thyroid cancer, such as obesity, smoking, race etc.^10^ Moreover, the EAPC of deaths showed a decrease trend in most of Sub‐Saharan Africa regions. Studies indicated that non‐Hispanic white individuals suffered with the highest incidence in the USA.^22^ In addition, non‐Hispanic black individuals had the lowest incidence, followed by Asian individuals and Pacific Islanders.^22^

Previous studies rare explored DALY of thyroid cancer. In our study, we found that the DALY increased, especially in middle SDI region and high‐income Asia Pacific. Because of over‐diagnosis and overtreatment, the thyroid cancer burden continued to increase. Moreover, the anxiety of thyroid cancer patients also led to overtreatment. In particular, patients with small thyroid nodules were more likely to be over‐treated, such as premature thyroidectomy, TSH suppression therapy, and Iodine 131 treatment. Actually, immediate surgery and watchful waiting might not be significantly different in thyroid cancer for preventing deaths.^23^ In addition, thyroid cancer was proved to be one of the tumors that most affected DALY.^24^ Thus, more targeted policy was urgent needed to reverse the rising trend caused by over‐diagnosis and overtreatment.

The risk factors of thyroid cancer have not been completely identified, and more risk factors are non‐modifiable, such as age, sex, race and family history. In our study, thyroid cancer was found to be more likely to occur in middle‐aged patients, and cause death in elderly patients. It might be related with the treatment improvement and aging of population.^25^ Obesity, ionizing radiation, and cigarette smoking were all proved to be related with the rising trend of thyroid cancer.^26^, ^27^ Among the risk factors, obesity was an important factor during the period.^27^ Additionally, high BMI was the only risk factor included in the GBD study of thyroid cancer. A study found that the trend was basically consistent with the trend in obesity prevalence. In addition, obesity had a greater impact on mortality than on incidence.^28^ Moreover, the aggressive clinical characteristics of thyroid cancer were proved to be associated with overweight and obesity via cross‐sectional studies.^29^ Further study also found that the aggressiveness and anaplastic change of thyroid cancer were directly affected by the diet‐induced obesity.^30^ In our study, we found that developed countries such as high‐income North American, Australasia, and Europe showed the highest proportion of deaths related with high BMI. Fortunately, the increase in obesity had slowed by recognizing the dangers of obesity in past decades. Moreover, the decline in obesity was particularly pronounced in developed countries.^31^ By paying attention to diet and weight control, the rising trend of thyroid cancer might be reversed in future.

Although we study the epidemiological characteristics, trend and risk factor of thyroid cancer. There were several limitations in our study. Due to the difference in the medical level, there may be missed diagnosis or over‐diagnosis in some areas. It may affect the accuracy of the GBD database and lead to misjudgment of the trend of thyroid cancer. Additionally, we could only study the risk factor included in GBD study. Among the risk factors for thyroid cancer, only high BMI was included in the GBD database. Furthermore, the association and interaction between risk factors cannot be studied with GBD data. By restrictions of data type, the GBD data of thyroid cancer was also not stratified by histologic characteristics. Thus, we could not discuss the difference among histologic characteristics in term of age, sex, region, and risk factor.

In summary, a rising trend of thyroid cancer was found via the GBD study. While recognizing the threat of thyroid cancer to our health, we also should recognize the burden caused by over‐diagnosis. It is also important to fully understand the risk factors of thyroid cancer. Moreover, it is of great significance to revise the guidelines for diagnosis and treatment of thyroid cancer and formulate targeted measures to reverse the rising trend of thyroid cancer.

## CONFLICTS OF INTEREST

The authors declare no conflicts of interest.

## AUTHORS’ CONTRIBUTIONS

Study design: Zhai MM, Long JH, and Li YM. Data collection: Zhang D, Long JH, and Liu SS. Data analysis: Zhang D, Gong Y, and Liu SS. Figures: Zhai MM and Ye F. Manuscript writing: Zhai MM, Long JH, and Liu SS. Manuscript proofing: Long JH, Zhai MM, and Li YM.

## Supporting information

Fig S1

Fig S2

Fig S3

Fig S4

Fig S5

## Data Availability

All date was available at http://ghdx.healthdata.org/gbd‐2017. As a noncommercial user of IHME websites, we followed the Creative Commons Attribution‐NonCommercial‐NoDerivatives 4.0 International License and Section 7 of the University of Washington's Website Terms and Conditions of Use in our study.
