# Supplementary figures and images for: The global burden of thyroid cancer and its attributable risk factor in 195 countries and territories: A systematic analysis for the Global Burden of Disease Study
Source: Cancer Med. 2021 May 18;10(13):4542–54. doi: 10.1002/cam4.3970 (PMC8267141; doi:10.1002/cam4.3970)

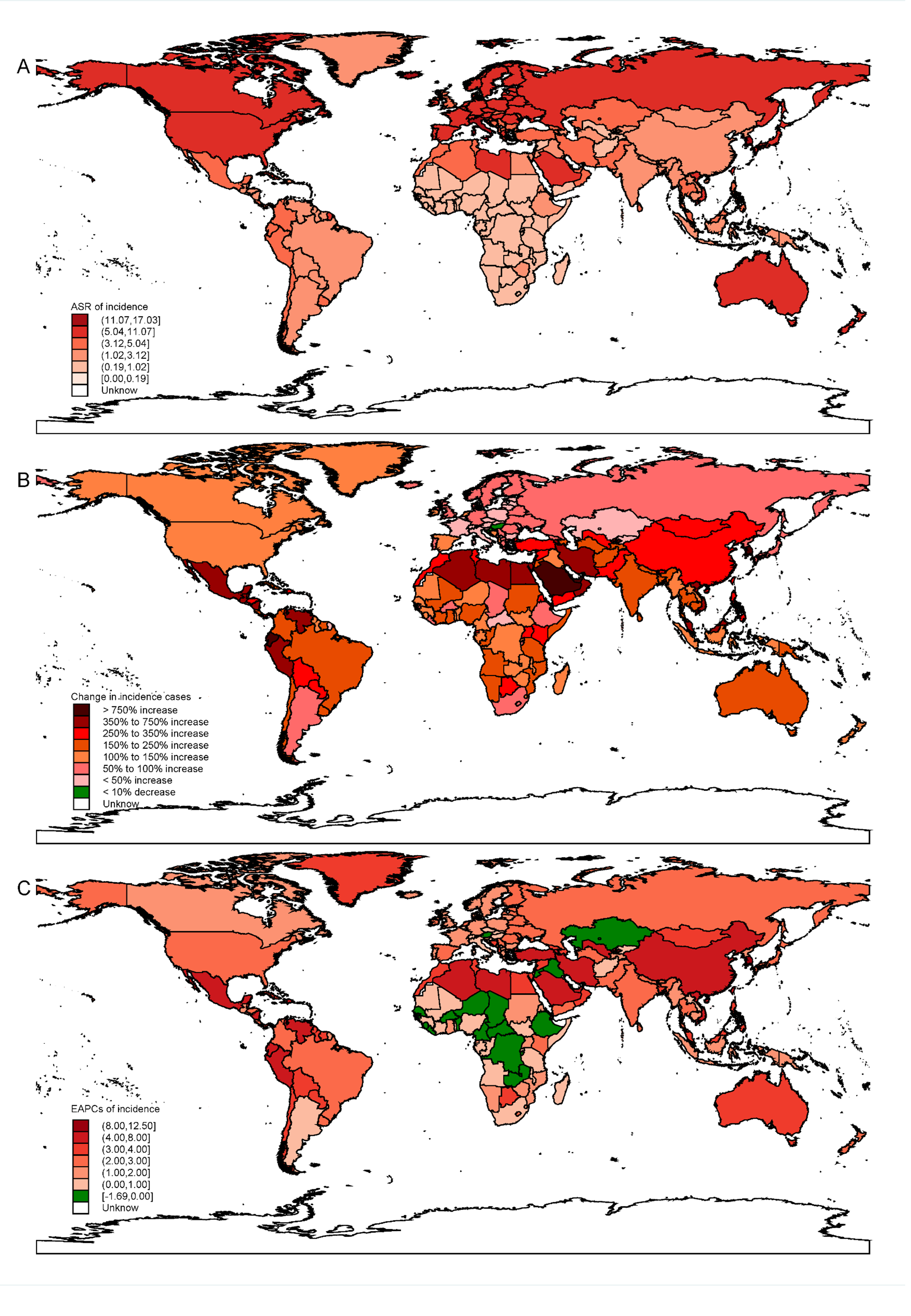

Supplement: Supplementary file 1 — Fig S1 [file CAM4-10-4542-s003.TIF]

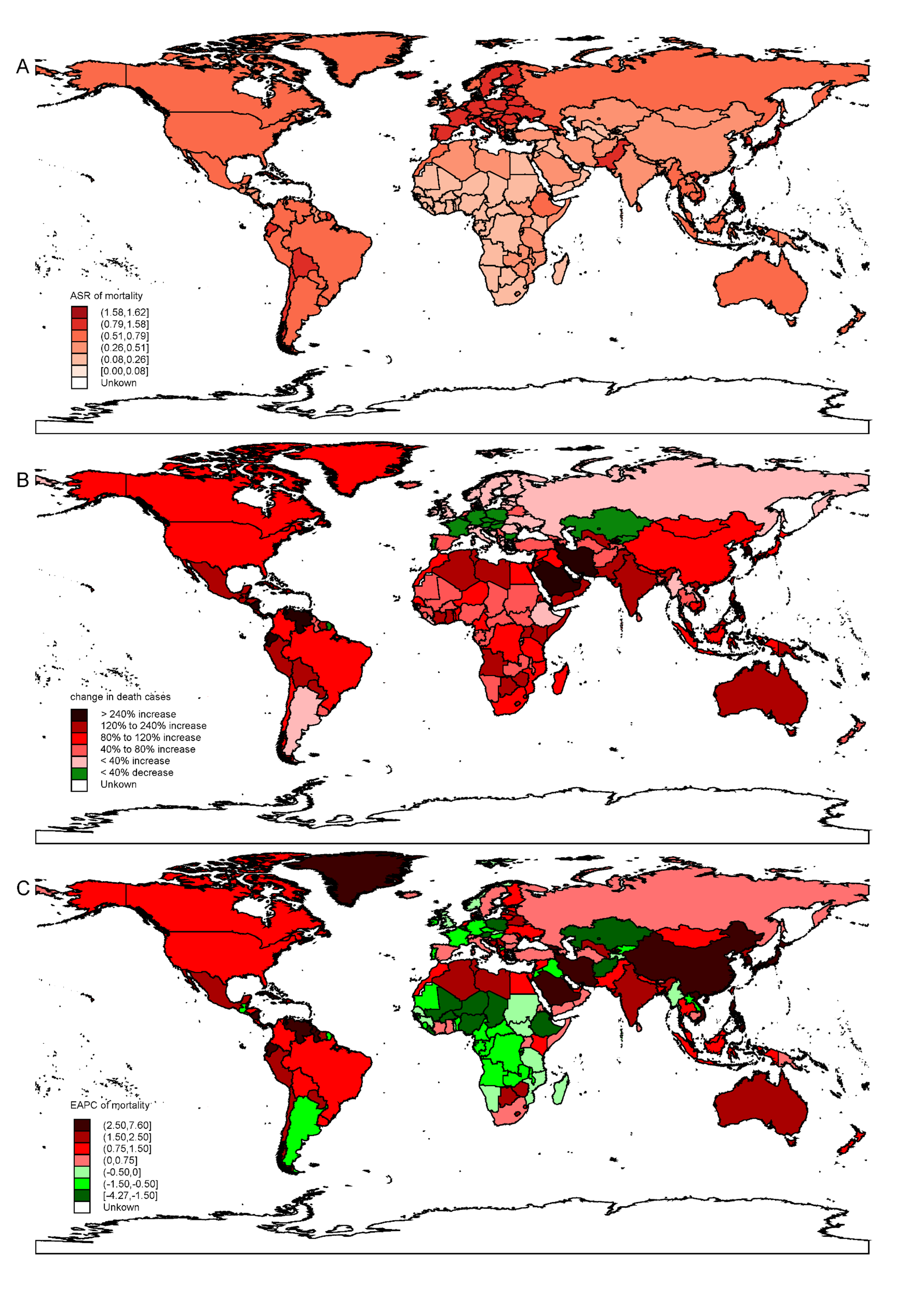

Supplement: Supplementary file 2 — Fig S2 [file CAM4-10-4542-s002.TIF]

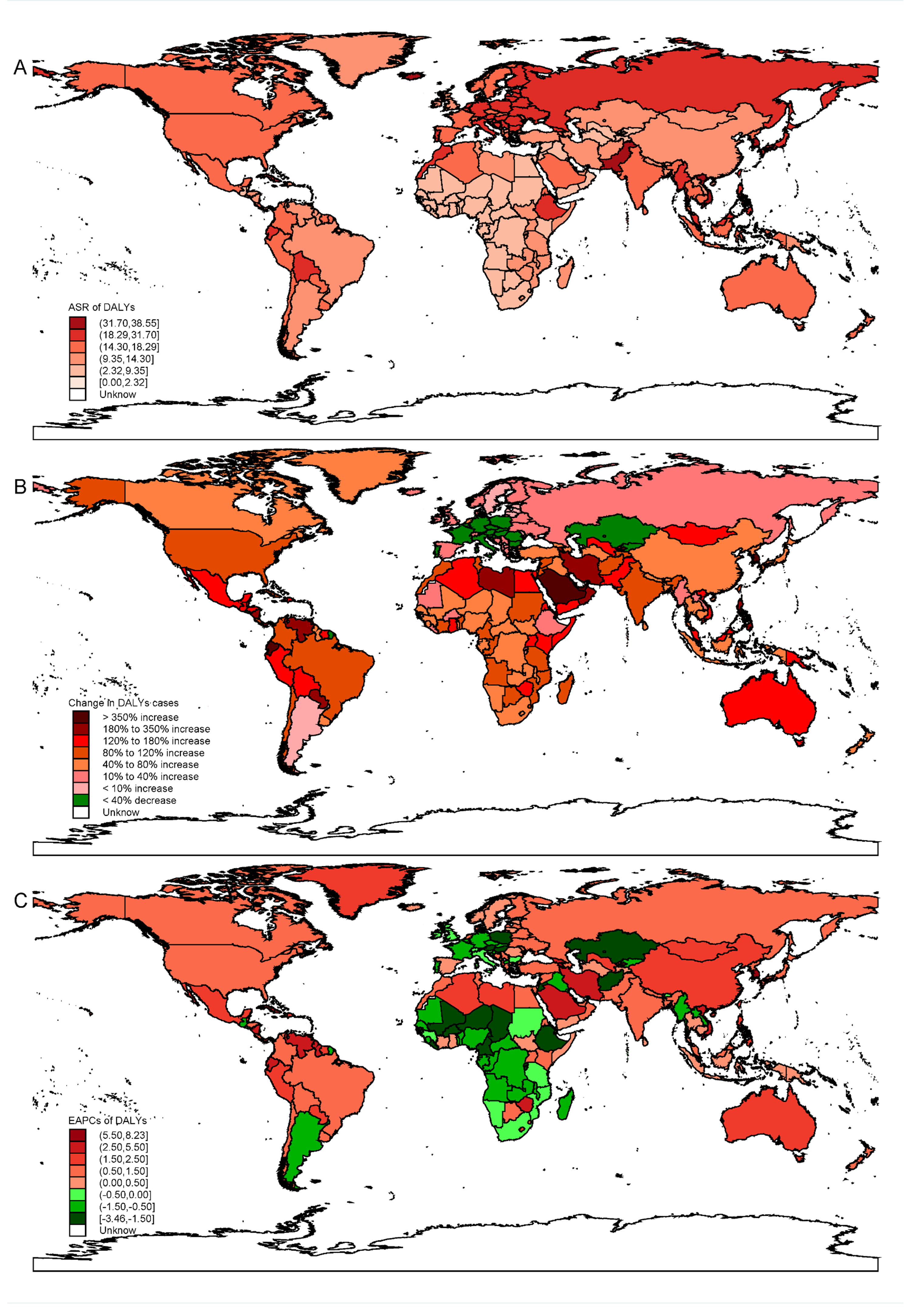

Supplement: Supplementary file 3 — Fig S3 [file CAM4-10-4542-s004.TIF]

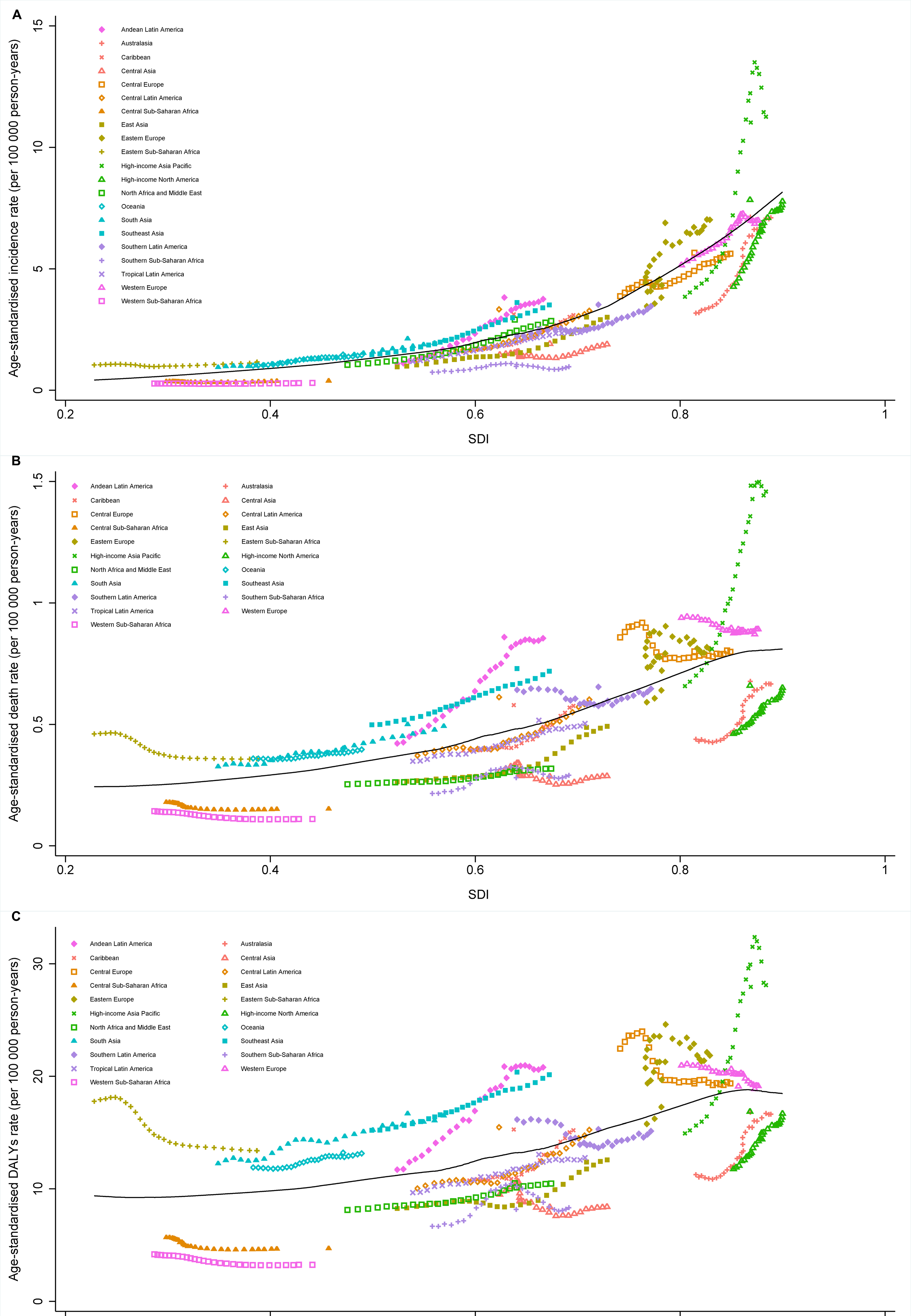

Supplement: Supplementary file 4 — Fig S4 [file CAM4-10-4542-s001.TIF]

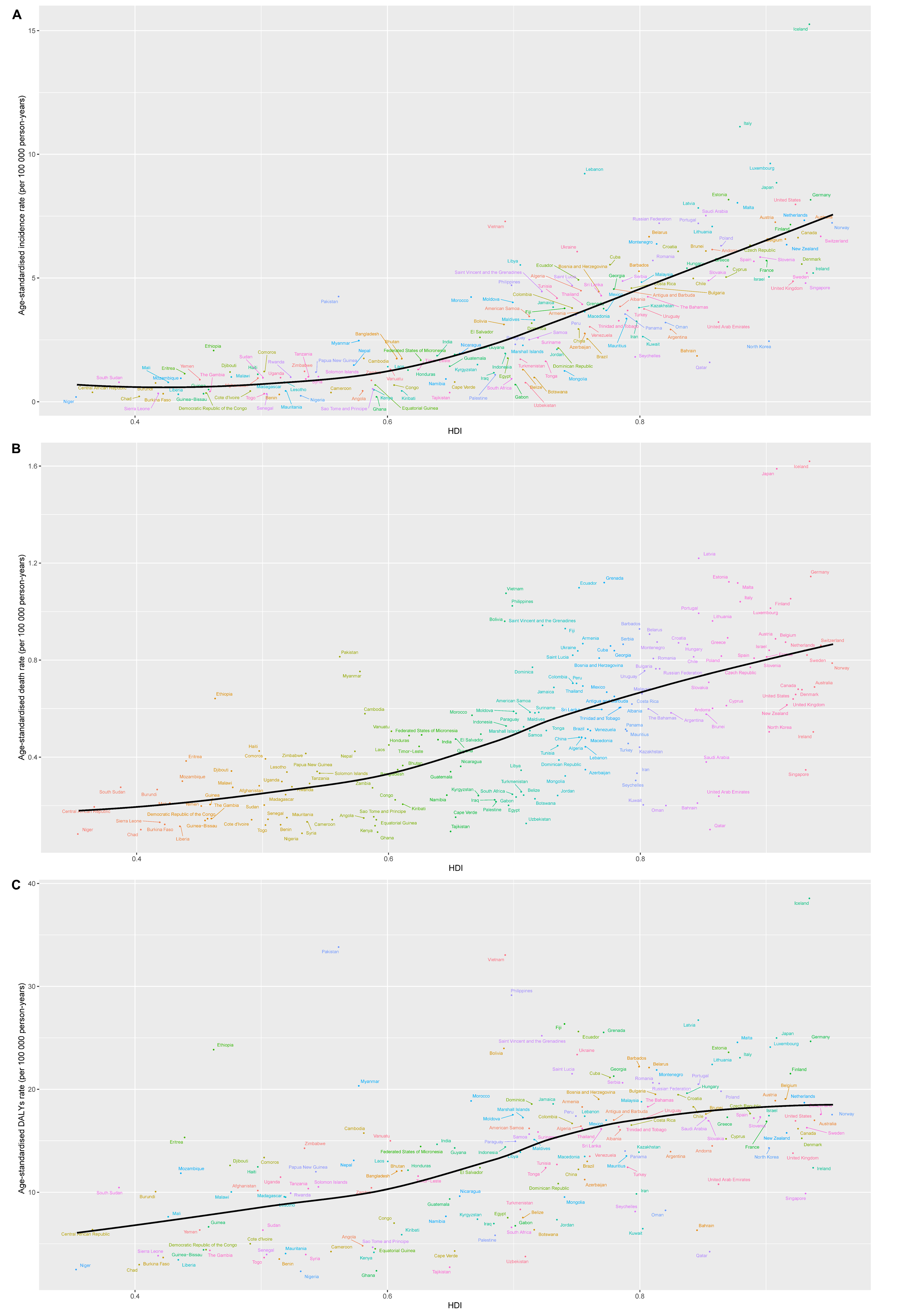

Supplement: Supplementary file 5 — Fig S5 [file CAM4-10-4542-s005.TIF]
